# Consciousness in Jawless Fishes

**DOI:** 10.3389/fnsys.2021.751876

**Published:** 2021-09-24

**Authors:** Daichi G. Suzuki

**Affiliations:** ^1^Graduate School of Life and Environmental Sciences, University of Tsukuba, Tsukuba, Japan; ^2^Center for Human Nature, Artificial Intelligence, and Neuroscience (CHAIN), Hokkaido University, Sapporo, Japan

**Keywords:** cyclostome, lamprey, ammocoetes, hagfish, minimal consciousness, primary consciousness

## Abstract

Jawless fishes were the first vertebrates to evolve. It is thus important to investigate them to determine whether consciousness was acquired in the common ancestor of all vertebrates. Most jawless fish lineages are extinct, and cyclostomes (lampreys and hagfish) are the sole survivors. Here, I review the empirical knowledge on the neurobiology of cyclostomes with special reference to recently proposed “markers” of primary, minimal consciousness. The adult lamprey appears to meet the neuroanatomical criteria but there is a practical limitation to behavioral examination of its learning ability. In addition, the consciousness-related neuroarchitecture of larvae and its reconstruction during metamorphosis remain largely uninvestigated. Even less is known of hagfish neurobiology. The hagfish forebrain forms the central prosencephalic complex, and the homology of its components to the brain regions of other vertebrates needs to be confirmed using modern techniques. Nevertheless, as behavioral responses to olfactory stimuli in aquariums have been reported, it is easier to investigate the learning ability of the hagfish than that of the lamprey. Based on these facts, I finally discuss the potential future directions of empirical studies for examining the existence of consciousness in jawless fishes.

## Introduction

**FIGURE 1:**
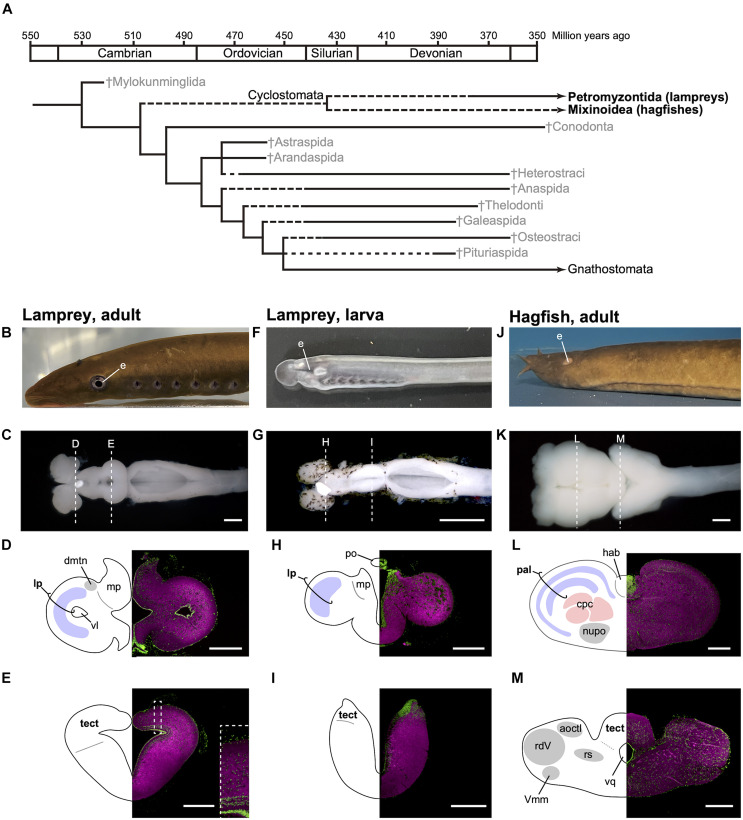
Phylogenetic tree of early vertebrates and brain sections of the cyclostomes. **(A)** Cladogram showing the postulated relationships of the jawless fishes and the Gnathostomata (jawed fishes) based on morphological characters (based on Benton, 2015). **(B–E)** Lateral view of adult lamprey (*Lethenteron camtschaticum*, **B**), dorsal view of the brain **(C)**, and its transverse brain sections at the forebrain **(D)** and midbrain **(E)** levels. The laminated structure of the optic tectum is magnified in the inset of **(E)**. **(F–I)** Lateral view of larval lamprey **(F)**, dorsal view of the brain **(G)**, and its transverse brain sections at the forebrain **(H)** and midbrain **(I)** levels. The photograph for **(G)** is reproduced from Suzuki and Grillner (2018). **(J–M)** Lateral view of adult hagfish (*Eptatretus burgeri*, **J**), dorsal view of the brain **(K)**, and its transverse brain sections at the forebrain **(L)** and midbrain **(M)** levels. Sections are immunostained by anti-acetylated tubulin antibody (Sigma, T6793, *magenta*) and counterstained with Fluorescent Nissl Stain (Invitrogen N21480, *green*). acoctl, area octavolateralis; cpc, central prosencephalic complex; dmtn, dorsomedial telencephalic nucleus; hab, habenular ganglion; lp, lateral pallium; mp, medial pallium; nupo, nuclei praeoptici; pal, pallium; po, pineal organ; tect, tectum; rdV, radix descendens nervi trigemini; rs, formation reticularis, pars superior; vl, ventriculus lateralis; Vmm, nucleus motorius magnocellularis nervi trigemini; vq ventriculus quartus. Scale bars: 1 mm for **(C,G,K)**; 500 μm for **(D,E,L,M)**; 200 μm for **(H,I)**.

The first vertebrates did not have a jaw. These jawless fishes (agnathans) prospered in the Paleozoic, but most of them went extinct (Figure 1A). Cyclostomes are the only extant agnathans, consisting of lampreys and hagfish. The jawed vertebrates (gnathostomes) evolved from one of these jawless lineages and then diverged. From a cladistic perspective, the terms “jawless fishes,” “jawless vertebrates,” and “agnathans” are invalid because they refer to a paraphyletic group. Nevertheless, I use these terms in here for convenience.

Until recently, it was thought that consciousness is limited to the animals with relatively high cognitive ability, such as mammals, birds, and perhaps cephalopods (e.g., Edelman et al., 2005; Edelman and Seth, 2009). However, various researchers have started to consider that all vertebrates, including fishes, share a basic type of consciousness, called primary consciousness or minimal consciousness (Feinberg and Mallatt, 2013, 2016, 2018; Brown, 2015; Bronfman et al., 2016; Ginsburg and Jablonka, 2019; Godfrey-Smith, 2016, 2020). If this is the case, the cyclostomes are important because they are the only remaining stem vertebrates.

Although lampreys and hagfish form a monophyletic group, their brain structures are distinct, reflecting their different lifestyles and lineage-specific adaptations (Figures 1B–M). It is thus important to note that modern cyclostomes possess both ancestral and derivative characters. Lampreys spend several years as filter-feeding ammocoetes larvae, which burrow in riverbeds. As the larva has immature eyes (Figure 1F), the optic tectum (the main visual center in non-mammalian vertebrates) also remains undeveloped (Figure 1I). On metamorphosis, the animal transforms into an active parasitic predator. Some lampreys are landlocked and breed soon after metamorphosis, while others migrate downstream to the sea or a large lake to attack their prey. The adult lamprey has well-developed eyes (Figure 1B) and a mature, layered optic tectum (Figure 1E). A recent study found that the lateral pallium of the lamprey has three layers, presumably representing the ancestral vertebrate state, from which the mammalian cortex is derived (Suryanarayana et al., 2017). In comparison, the hagfish undergoes direct development and has adapted to the deep sea, so its eyes and tectum are degenerate (Figures 1J,M). The hagfish forebrain is enlarged (Figure 1L) and predominantly receives olfactory input (Wicht and Northcutt, 1993).

In this paper, I explore the current empirical knowledge on the neurobiology of cyclostomes in light of the evolution of consciousness. First, I briefly describe recently proposed “markers” of primary, minimal consciousness. Then, I review current empirical knowledge on the neurobiology of lampreys and hagfish, examining the extent to which the existence of the “markers” is supported in these organisms. Lastly, I discuss possible directions for further studies of consciousness in jawless fishes.

## “Markers” of Primary, Minimal Consciousness

Among recently proposed accounts of the evolution of consciousness, the theories of Feinberg and Mallatt (2016, 2018), and Ginsburg and Jablonka (2019) are the most detailed and supported by abundant empirical data. In discussing the evolutionary origin of consciousness, the authors use different “markers” of consciousness, while their conclusions are the same; they agree that all vertebrates, as well as some arthropods (including insects) and cephalopods (possibly only coleoids), have consciousness. In this section, I briefly review the two theories and the “markers” of consciousness suggested by these authors.

Feinberg and Mallatt (2016, 2018) distinguish two major aspects of consciousness, exteroceptive and affective consciousness; interoceptive consciousness is intermediate to the two (Feinberg and Mallatt, 2018, Figure 2.4). Their criteria for the exteroceptive consciousness consist of several “special” neurobiological features; complex neural hierarchies (i.e., true brains), isomorphic representations (e.g., somatotopy and retinotopy), multimodal integration (“nested and non-nested hierarchical functions” in their words), interregional neural interactions, and attention. The neuroanatomical and behavioral criteria for affective consciousness include operant learning involving global affective responses and relevant reward/punishment systems [e.g., the ventral tegmental area (VTA) and habenular nucleus].

In contrast to the enumerative approach of Feinberg and Mallatt (2016, 2018), and Ginsburg and Jablonka (2019) argue that a form of associative learning, which they call “unlimited associative learning (UAL),” is *the* positive marker of consciousness. UAL requires a list of capacities (e.g., global accessibility, binding, selective attention, evaluative system, and agency) that suffice for being conscious (Birch et al., 2020). Lacking clear evidence for UAL, they also admit “proxies,” including Pavlovian conditioning with compound conditional stimuli, operant conditioning involving novel action patterns, conceptual learning, and navigation learning (Ginsburg and Jablonka, 2019, Table 8.1).

These criteria for consciousness raise two questions. How many of the features listed in the criteria of consciousness proposed by Feinberg and Mallatt (2016) do lampreys and hagfish possess, and do the cyclostomes show UAL or its proxies? In the following sections, I examine these questions applying available empirical evidence.

## Lamprey

The adult lamprey has been used as an experimental model for investigating the basic neuroarchitecture of vertebrates (Grillner et al., 1998; Auclair and Dubuc, 2020), and its neurobiology is relatively well-known. Feinberg and Mallatt (2016) use this knowledge to discuss whether the lamprey has consciousness based on their criteria (pp. 104–115). Current neurobiological findings in fact indicate that the lamprey meets their criteria for exteroceptive consciousness as follows (see also Table 1). First, the lamprey brain shares basic brain regions (i.e., the telencephalon, diencephalon, mesencephalon, cerebellum, and rhombencephalon) and developmental mechanisms with other vertebrates (Pombal and Puelles, 1999; Murakami et al., 2001; Pombal et al., 2009; Sugahara et al., 2011, 2016; Murakami, 2017). Second, the optic tectum has a laminar structure, of which the superficial layer receives visual input with retinotopy (Jones et al., 2009). Third, electroceptive inputs are sent to the intermediate layer with spaciotopy, being integrated with visual perception (Kardamakis et al., 2016). In addition, retinotopic and somatotopic organization is found in the lateral portion of the pallium (a telencephalic structure homologous to the mammalian cortex) (Suryanarayana et al., 2020). The lateral pallium sends output to the optic tectum (Ocaña et al., 2015), while the optic tectum sends its fibers to the thalamus (Northcutt and Wicht, 1997), which is the relay center between the pallium/cortex and other brain regions. This suggests that there is a mutual interaction between the pallium and optic tectum (Suzuki and Grillner, 2018, Figure 1C). Lastly, the optic tectum also has mutual connections to the SNc/VTA (SNc: substance nigra pars compacta), which detects the saliency of the visual stimuli and returns the information to the optic tectum via dopaminergic axons (Pérez-Fernández et al., 2017).

**TABLE 1:** The criteria of consciousness and neurobiological evidence in the cyclostomes.

	Lamprey, Adult		Lamprey, Larva		Hagfish	
Feinberg and Mallatt (2016)						
**Exteroceptive consciousness**						
Complex neural hierarchy (true brain)	*Yes*	Murakami, 2017; Murakami et al., 2001; Sugahara et al., 2011, 2016	*Yes*	Murakami et al., 2001; Murakami, 2017	*Yes*	Larsell, 1947, 1967; Murakami, 2017
		Pombal and Puelles, 1999		Sugahara et al., 2011, 2016		Sugahara et al., 2016, 2017
		Pombal et al., 2009				
Isomorphic representations	*Yes*	Jones et al., 2009; Kardamakis et al., 2016	n.d.	−	*Yes ?*	Amemiya, 1983; Nishizawa et al., 1988
Multimodal integration	*Yes*	Kardamakis et al., 2016	n.d.	−	*Yes ?*	Ronan, 1988; Ronan and Northcutt, 1990; Wicht and Nieuwenhuys, 1998
Interregional neural interaction	*Yes*	Northcutt and Wicht, 1997; Ocaña et al., 2015	n.d.	−	n.d.	−
Attention	*Yes*	Pérez-Fernández et al., 2017	n.d	−	n.d.	−
**Affective consciousness**						
Operant learning involving global affective response	n.d.	−	n.d.	−	n.d.	−
The relevant reward/punishment system (e.g., VTA, habenular nucleus)	*Yes*	Stephenson-Jones et al., 2011, 2012; Pérez-Fernández et al., 2017; Grillner et al., 2018	n.d.	−	n.d.	−
Ginsburg and Jablonka (2019)						
UAL or its proxies	n.d.	−	n.d.	−	n.d.	−
						

*n.d., not determined.*

Regarding affective consciousness, the lamprey possesses the neuroarchitecture for reward/punishment systems. For example, dopaminergic neurons in the SNc/VTA region send axons not only to the optic tectum (as mentioned above) but also to the basal ganglia, which presumably contributes to reward prediction and motor decision-making based on the prediction (Stephenson-Jones et al., 2011; Pérez-Fernández et al., 2017). The lateral habenula is also present and probably contributes to the reward coding and aversive behavior (Stephenson-Jones et al., 2012; Grillner et al., 2018). The medial habenula sends projections to the interpeduncular nucleus (IPN) and further to the PAG/griseum centrale (PAG: periaqueductal gray) and is perhaps mediates freezing and flight responses (Stephenson-Jones et al., 2012; Grillner et al., 2018). However, little behavioral research has examined learning in the lamprey due to the practical limitation that available adult lampreys are postmetamorphic juveniles before downstream migration or mature upstream-migrated fish, both of which lack appetites, making them unsuitable for learning experiments using food rewards. Notably, anadromous adult lamprey can only be alpha conditioned [i.e., conditioning that is based on habituated unconditional stimuli (USs)] and do not show true Pavlovian conditioning when strong lights, strong electric shocks, and nocuous tactile stimulations are used as USs, and weak lights, mild shocks, mild tactile stimuli, sounds, and odors are used and conditional stimuli (CSs) preceding the USs by 3–5 s (Sergeyev, 1964; Razran, 1971).

Interestingly, the lamprey brain changes drastically during postembryonic development. The larval tectum remains immature and becomes laminated during metamorphosis, as mentioned above. The primary retina, which forms during embryogenesis, is also immature and thought to function in non-directional or broadly directional photoreception (Villar-Cerviño et al., 2006; Suzuki et al., 2015a,b; Suzuki and Grillner, 2018). The primary optic nerve projects not to the optic tectum but to the diencephalic pretectum (Suzuki et al., 2015a). A similar neural organization for photoreception is found in amphioxus (Suzuki et al., 2015a), which is a close invertebrate relative of vertebrates and judged to be non-conscious based on the criteria of Feinberg and Mallatt (2016). There are differences in the cytological architecture (discussed in Suzuki et al., 2015a), suggesting a need to analyze the origin of the vertebrate visual system in terms of cell type evolution, possibly with reference to genome duplication in the vertebrate lineage. Nonetheless, the architectural similarity between the two groups implies that the lamprey larval neural circuits for photoreception represent an ancestral state before the evolution of image-forming vision. The marginal region of the primary retina expands into the secondary retina during the entire larval period. The retinal ganglion cells in this secondary retina differentiate before metamorphosis, and the secondary optic nerve projects to the optic tectum with retinotopy (Cornide-Petronio et al., 2011), whereas other retinal cell types (the photoreceptors, horizontal calls, and amacrine cells) differentiate during metamorphosis (De Miguel et al., 1989; Pombal et al., 2003; Villar-Cerviño et al., 2006; Abalo et al., 2008). Thus, the image-forming vision established by the optic tectum is actualized only after the metamorphosis (Suzuki and Grillner, 2018; Suzuki et al., 2019). These findings suggest that the consciousness-related neural circuits are immature during the larval stage and are then reconstructed into the full-blown, functional neuroarchitecture for consciousness during metamorphosis. In other words, the lamprey may undergo transformation from a non-conscious larva to a conscious adult (Suzuki and Grillner, 2018).

Furthermore, the similarity of the neural organization for photoreception between the amphioxus and lamprey larvae implies parallelism between the developmental transformation in the lamprey and the evolutionary transformation in the vertebrate lineage from non-conscious to conscious. However, a recent fossil study indicated that stem lampreys lacked the ammocoetes larval stage (Miyashita et al., 2021), suggesting that the metamorphosis of modern lampreys was acquired secondarily. Evans et al. (2018) agree that ancestral lampreys were direct developers and propose a “condensation hypothesis,” which holds that stem lampreys possessed both modern larval and juvenile characters. Differential selection favored segregation of the larval characters in the beginning of the life history and juvenile characters after, requiring metamorphosis to accommodate such body reconstruction. If this is the case, it is possible that stem lampreys gradually developed derivative consciousness-related brain structures, including an image-forming visual system, without evident metamorphosis. Then the development of those structures was condensed in later stages, accompanied by the acquisition of metamorphosis. In either case, the relationship between the evolutionary origin of vertebrate consciousness and the development of lamprey consciousness is an intriguing research topic in terms of evolutionary developmental (evo-devo) biology. Nevertheless, the neural circuits in the larval brain and their transformation during metamorphosis, especially of the optic tectum, remain largely uninvestigated and need further study. The learning ability of the ammocoetes larva is also unknown.

Therefore, the adult lamprey meet the criteria of Feinberg and Mallatt (2016) for exteroceptive consciousness. For affective consciousness, the neuroanatomical criteria are satisfied, although behavioral evidence is lacking. The existence of UAL or its proxies has not been confirmed, thus not meeting the requirement of Ginsburg and Jablonka (2019). The larval lamprey does not appear to satisfy any of the criteria described above, although much more study is needed. If in fact the lamprey changes from non-conscious to conscious during metamorphosis, studies of this transformation will provide valuable information about both the development and evolution of consciousness.

## Hagfish

Much less is known about the neurobiology of the hagfish than that of the lamprey. Although a recent developmental study revealed that the developmental mechanisms underlying formation of the forebrain are conserved in the hagfish (Sugahara et al., 2016), the hagfish forebrain later forms the central prosencephalic complex, and the homology of its components to the brain regions of other vertebrates is unclear (Wicht and Nieuwenhuys, 1998). As a hagfish-specific character, there is no overt epiphysis. A morphologically distinct cerebellum is also absent, while developmental genes involved in cerebellum formation (*Pax6* and *Atoh1*) are expressed in the rhombic lip, from which the cerebellum differentiates (Sugahara et al., 2016, 2017). At the posterior end of the midbrain, there is a portion of the acousticolateral (or vestibulolateral) commissure, which can be regarded as the rudimentary cerebellum (Larsell, 1947, 1967; Sugahara et al., 2017). These findings suggest that the common ancestor of vertebrates possessed at least a non-layered simple cerebellum, similar to that of lampreys.

As mentioned above, the hagfish has degenerate eyes due to adaptation to the deep sea. Fossil evidence indicates that this is a secondary modification specific to the hagfish lineage (Gabbott et al., 2016). In concordance with the degeneration of the eyes, the retinotectal projection is largely reduced, and the retinopretectal pathway becomes dominant (Kusunoki and Amemiya, 1983; Wicht and Northcutt, 1990). Despite no empirical evidence, the degenerate state of the eyes and retinotectal projection implies no or severely disorganized retinotopy in the tectum. Still, it receives inputs from various regions responsible for different sensory modalities (e.g., the octavolateral area, sensory nucleus of the trigeminal nerve, and dorsal column nuclei), suggesting that it functions as an integrative center (Amemiya, 1983; Ronan, 1988; Ronan and Northcutt, 1990; Wicht and Nieuwenhuys, 1998). Furthermore, primary trigeminal afferents are arranged somatotopically in the sensory nucleus of the trigeminal nerve according to the ramus in which they are distributed toward the periphery (Nishizawa et al., 1988). It remains to be determined whether this somatotopic organization is maintained in the tectum. In addition, the hagfish has peculiar taste bud-like chemosensory organs, the Schreiner organs, which are distributed throughout the epidermis and in the prenasal sinus, nasopharyngeal duct, and pharynx at high densities, and in the oral and velar chambers at lower densities (Braun, 1998). These organs are innervated by the trigeminal and glossopharyngeal/vagal nerves and the cutaneous rami of spinal nerves (Braun, 1998). It is plausible that the mechanosensory and chemosensory perception are initially segregated in the primary receptive areas and they are integrated with each other and inputs from other sensory modalities in a higher integrative center. One possibility is that the chemosensory inputs from the Schreiner organs are also received by the tectum. However, these postulates lack solid empirical evidence.

The most prominent sensory modality in the hagfish is olfaction. Its main brain center is the pallium, the forebrain region homologous to the mammalian cortex (Wicht and Northcutt, 1993). The hagfish pallium consists of five layers (Jansen, 1930; Wicht and Northcutt, 1992). Recently, Suryanarayana et al. (2017, 2021) revealed that the lamprey has three layered cortices, which share neuroanatomical and neurophysiological features with those of the reptiles, perhaps being a precursor of the mammalian six-layered neocortex. However, no molecular studies have examined layer-specific genes. Expression analysis on the layer-specific genes is required to elucidate the evolutionary relationships between the five hagfish and three lamprey layers (i.e., which hagfish and lamprey layers correspond), and between the three lamprey layers and the three reptile layers (i.e., whether they are truly homologous or just convergent).

Despite the patchy information, the above findings suggest that the hagfish satisfies some features listed in the criteria of Feinberg and Mallatt (2016) for exteroceptive consciousness (Table 1). However, many of the consciousness-related neuroanatomical features remain to be investigated, including the attention and affective systems.

Still, the hagfish appears to have an advantage in behavioral experiments over the lamprey because it will feed in an aquarium. Recently, Glover et al. (2019) reported that the chemosensory behavior of the hagfish can be assessed using a modified T-maze arena, in which food or noxious stimuli are placed in one of the arms of the maze. This suggests that hagfish learning behavior can be investigated using food as a reward. The degenerate vision of the hagfish is a disadvantage in designing learning experiments. However, odor, taste, and tactile stimuli can be combined to apply compound stimuli, which are required for UAL or its proxies.

## Conclusion and Future Directions

The cyclostomes are the sole surviving jawless fishes, which were the first vertebrates to evolve. To examine the existence of consciousness in jawless fishes, I assessed knowledge on the neurobiology of the cyclostomes, i.e., lampreys and hagfish, while referring to recently proposed criteria for animal consciousness. The neuroanatomy of the adult lamprey meets the criteria of Feinberg and Mallatt (2016) for exteroceptive consciousness, but much information is lacking.

First, the learning behavior of the adult lamprey needs to be investigated to determine whether the criteria of Feinberg and Mallatt (2016) for affective consciousness are satisfied and whether UAL or its proxies (Ginsburg and Jablonka, 2019) are observed. For this purpose, an innovative experimental design is needed, since available adults do not show appetitive behavior in an aquarium.

Second, the consciousness-related neural circuits in the larval brain and their transformation during metamorphosis, as well as the learning ability of the larva, will be an intriguing subject from the evo-devo perspective on consciousness. Establishment of the multimodal isomorphic (e.g., retinotopic and electroceptive spatiotopic) organization of the optic tectum is of special interest.

Lastly, the neurobiology of the hagfish is less developed in terms of neuroanatomy, neurophysiology, and neuroethology. Further studies using modern approaches, such as gene expression analysis, would improve our understanding of this mysterious creature.

To conclude, we have patchy knowledge on the neurobiology of the cyclostomes for discussing the consciousness of jawless fishes. Despite taxon-specific difficulties in their investigation, further effort is required to elucidate the early evolution of consciousness in the vertebrate lineage.

## Data Availability Statement

The original contributions presented in the study are included in the article/supplementary material, further inquiries can be directed to the corresponding author.

## Author Contributions

DS wrote the manuscript.

## Conflict of Interest

The author declares that the research was conducted in the absence of any commercial or financial relationships that could be construed as a potential conflict of interest.

## Publisher’s Note

All claims expressed in this article are solely those of the authors and do not necessarily represent those of their affiliated organizations, or those of the publisher, the editors and the reviewers. Any product that may be evaluated in this article, or claim that may be made by its manufacturer, is not guaranteed or endorsed by the publisher.
